# Chemoresistance is mediated by ovarian cancer leader cells in vitro

**DOI:** 10.1186/s13046-021-02086-3

**Published:** 2021-09-01

**Authors:** Nazanin Karimnia, Amy L. Wilson, Emma Green, Amelia Matthews, Thomas W. Jobling, Magdalena Plebanski, Maree Bilandzic, Andrew N. Stephens

**Affiliations:** 1 Hudson Institute of Medical Research, 27-31 Wright St, Clayton, VIC 3168 Australia; 2 Department of Molecular and Translational Sciences, Monash University, Clayton, Australia; 3 Monash Medical Centre, Department of Gynaecology Oncology, Monash Health, Moorabbin, Australia; 4 School of Health and Biomedical Sciences, RMIT University, Bundoora, Australia

**Keywords:** Ovarian Cancer, Leader cells, Keratin-14, Chemo-resistance, Recurrence

## Abstract

**Background:**

Leader cells are a subset of cancer cells that coordinate the complex cell-cell and cell-matrix interactions required for ovarian cancer migration, invasion, tumour deposition and are negatively associated with progression-free survival and response to therapy. Emerging evidence suggests leader cells may be enriched in response to chemotherapy, underlying disease recurrence following treatment.

**Methods:**

CRISPR was used to insert a bicistronic T2A-GFP cassette under the native KRT14 (leader cell) promoter. 2D and 3D drug screens were completed in the presence of chemotherapies used in ovarian cancer management. Leader cell; proliferative (Ki67); and apoptotic status (Cleaved Caspase 3) were defined by live cell imaging and flow cytometry. Quantitative real-time PCR defined “stemness” profiles. Proliferation was assessed on the xCELLigence real time cell analyser. Statistical Analysis was performed using unpaired non-parametric t-tests or one-way ANOVA and Tukey’s multiple comparison post hoc.

**Results:**

Leader cells represent a transcriptionally plastic subpopulation of ovarian cancer cells that arise independently of cell division or DNA replication, and exhibit a “stemness” profile that does not correlate with epithelial-to-mesenchymal transition. Chemotherapeutics increased apoptosis-resistant leader cells in vitro, who retained motility and expressed known chemo-resistance markers including *ALDH1*, *Twist* and *CD44v6*. Functional impairment of leader cells restored chemosensitivity, with leader cell-deficient lines failing to recover following chemotherapeutic intervention.

**Conclusions:**

Our data demonstrate that ovarian cancer leader cells are resistant to a diverse array of chemotherapeutic agents, and are likely to play a critical role in the recurrence of chemo-resistant disease as drivers of poor treatment outcomes.

**Supplementary Information:**

The online version contains supplementary material available at 10.1186/s13046-021-02086-3.

## Background

Ovarian cancer is the most lethal of all gynaecological malignancies and the seventh most commonly diagnosed cancer amongst women [1]. Characterised by its asymptomatic progression, over 75% of patients are diagnosed with late stage disseminated disease involving multiple tumor deposits and ascites upon primary presentation [2]. First line treatment options involve surgical debulking and combination platinum and taxane-based chemotherapy, to which most patients initially respond encouragingly [2]. However, over 90% of ovarian cancer patients develop recurrent, platinum-resistant disease, limiting therapeutic options and underlying the high mortality rate [3]. There has been minimal improvement to ovarian cancer survival rates since the introduction of platinum-based chemotherapy over 30 years ago [4].

Ovarian cancer remains one of the most difficult cancers to detect and treat, in part due to its unique mode of dissemination as heterogeneous, anchorage-independent spheroids [2]. Ovarian cancer spheroids display inherent chemoresistance, and seed multiple distal metastases throughout the peritoneal cavity. Spheroids invade and migrate as a cohesive unit in a directed, coordinated fashion, in a process termed collective invasion [5]. Collective invasion is a fundamental but poorly characterised process common to many metastatic epithelial tumours [6–10].

A key mediator of collective invasion are Leader Cells (LCs), a subset of cancer cells that coordinate the complex cell-cell and cell-matrix interactions required to establish new tumour deposits [3, 4]. In previous work we identified the LC subset as essential for ovarian cancer migration and invasion [11]. LCs also possess features reminiscent of cancer stem cells (CSCs) including self-renewal, tumor initiation and a high degree of transcriptional plasticity, making them distinct from cells undergoing epithelial-to-mesenchymal transition (EMT) and the bulk “follower cell” population [10, 12, 13]. Whilst “CSCs” are enriched following first line ovarian cancer treatments, a consensus on the nature and markers defining CSCs in ovarian tumors has not been reached and is likely masked by the highly heterogeneous nature of the disease [14]. Analogous to CSCs, the LC subset is enriched in response to chemotherapy and may mediate disease recurrence following treatment with chemotherapeutics in bladder cancer [12]. Furthermore, our own studies implicated the basal epithelial marker KRT14 as an absolute determinant for OC spheroid integrity, mesothelial attachment and invasive potential, whereby KRT14 loss abrogated OC invasive capacity in vitro, with no affect on cell viability or proliferation [11]. Moreover, we demonstrated increased KRT14 expression is negatively associated with progression-free survival and response to therapy in ovarian cancer patients [11].

In this study we have investigated the nature of LCs in the context of stem-like gene expression and response to chemotherapy. Our data show that LCs are not a fixed lineage of quiescent cells, with a high degree of transcriptional plasticity that respond to environmental stimuli. LCs also displayed substantial resistance to a diverse array of chemotherapies, including those commonly used as first- and second-line therapies, suggesting LCs may contribute significantly to acquired chemoresistance and influence overall treatment outcomes.

## Methods

### Cell culture

We utilized the high grade serous and clear cell carcinoma ovarian cancer cell lines: SKOV3, ATCC (ATCC® #HTB-77™); COV362.4, Sigma Aldrich (Sigma #07071904) and OVCAR4, National Cancer Institute (RRID:CVCL_1627). Cells were maintained in: SKOV3 Dulbecco’s Modified Eagle Medium (DMEM)/Ham’s F-12 (DMEM/F12) (Thermo Scientific, #11965118); COV362.4 High glucose Dulbecco’s Modified Eagle Medium (DMEM-HG); and OVCAR4 RPMI-1640 (with sodium bicarbonate; Sigma-Aldrich, #R0883). Media was supplemented with 10% Fetal Calf Serum (FCS) (Thermo Fisher, #16000044) and 1% Penicillin-Streptomycin (Thermo Scientific, #15240062) with all lines maintained at 37 °C with 5% CO_2_. Cell lines were verified and authenticated by the Australian Genome Research Facility Ltd. Cell counts were conducted prior to experimentation by Trypan Blue viability staining using the Countess® II viable cell count system, cells routinely tested negative for mycoplasma. 3D spheroids were formed by seeding cells into Corning® spheroid Ultra-low adhesion microplates (Sigma-Aldrich #CLS4520) with spheroid integrity, size and shape monitored by live cell microscopy to ensure uniformity across experiments. KRT14 overexpressing and knockout cell lines (LC^OE^/ LC^KO^) were generated according to the previously described methods [11].

### T2A-GFP construct generation

SnapGene molecular biology software (version 4.2) was used to design a HR130PA vector (BioCat #HR130PA-1SBI), containing a T2A-GFP construct, a puromycin selection cassette and an RFP marker. A 5′ homology arm (HA) was designed complimentary to 0.5-1 kb upstream of the KRT14 stop codon. The HA was designed to remove the endogenous KRT14 stop codon and facilitate an in-frame junction between the KRT14 gene and the T2A-GFP construct. The 3′ HA was designed to be complementary to 0.5-1 kb of the 3’UTR, downstream of the guide strand (GS) target cleavage site. Each arm was inserted into the HR130PA vector, flanking the T2A-GFP cassette. The vector was synthesised by Genewiz. ChopChop web tool (www.chopchop.cbu.uib.no) was used to identify Cas9 target sites in the 3’UTR of KRT14. Four GS target sites were chosen and synthesised based upon the ranked list generated by ChopChop and cloned into the CRISPR Cas9 transfer vector lentiCRISPR V1 (Addgene #52961). CRISPR-mediated integration was performed as per the the HR130PA vector (BioCat #HR130PA-1SBI) protocol. Cells were co-transfected with the guide strand containing lentiCRISPR V1 transfer vector and the HA template using Lipofectamine® 2000 Transfection Reagent (Invitrogen, #11668019) in serum-free DMEM as per the manufacturers protocol, at a 2:1 ratio of homology repair template to transfer vector. Following transfection and a 12-h recovery period, cells were sub-cultured into selective medium and maintained under selective pressure by the addition of 1 μg/ml puromycin (Sigma-Aldrich, #P8833), as empirically determined by kill curves. The selection medium was replaced every 2 days for approximately 2 weeks and cells were subsequently subject to single cell sorting by flow cytometry based upon GFP^+^/RFP^+^ status. Individual colonies were expanded and target integration (homozygous or heterozygous) was verified by genomic PCR and Sanger sequencing (MHTP Medical Genomics Facility, Clayton, Australia).

### Flow cytometry of GFP^+^ LCs

A total of one million T2A-GFP cells from each line were collected into sterile 50 mL tubes, resuspended in 1% FBS in PBS and passed through filtered FACS tubes (Corning #352003). Cells were sorted on BD FACSAria™ Fusion/II (BD Biosciences) as a single positive leader (RFP^+^/GFP^+^) or follower (RFP^+^/GFP^−^) cell into a single well of a half area 96-well plate (Greiner Bio-One #675090) and imaged regularly using the Cytation3 multimode reader (Biotek). Untransfected wild-type cells served as negative controls for gating purposes.

### CellTrace™ blue cell proliferation

The CellTrace™ Blue cell proliferation kit (ThermoFisher, #C34568) was utilized to label 1 × 10^6^ cells as per manufacturers protocol by incubation with 5 μM CellTrace™ Blue in protein- free medium for 20 min at 37 °C. Cells were washed with culture medium containing 1% FCS and seeded at a low density into replicate wells and maintained at 37 °C with 5% CO_2_. CellTrace™ Blue fluorescence (355_ex_ /410_em_) and LC status GFP^+^/RFP^+^ was routinely assessed by microscopy using the Cytation™ 3 Multimode Imager (Biotek).

### Chemotherapeutic treatments and tracing the LC population by fluorescence microscopy

LC-T2A-GFP lines were seeded at 1200 cells per well of a 384-well plate, adhered overnight and treated with chemotherapeutics olaparib, rucaparib, paclitaxel, topotecan, carboplatin, cyclophosphamide, cisplatin and doxorubicin (Selleck) at empirically determined clinically relevant doses (1 μM). Cells were maintained at 37 °C with 5% CO_2_ in treatment for 72 h. Four focal areas per replicate well were imaged using 4X magnification on bright field, RFP and GFP using the Cytation™ 3 imaging system (Biotek) maintained at 37 °C and 5% CO_2_. Images were analysed as detailed below (Cytation™ 3 Multimode Imaging).

### Cytation™ 3 multimode imaging

Images were captured at 4X magnification using the Cytation™ 3 Multimode Imager, equipped with Gen5 Image prime software (V3.04 Biotek) and standard imaging filter/LED cubes for RFP/GFP/DAPI or as indicated. 37 °C with 5% CO_2_ was maintained during live cell imaging experiments. Automated settings were used to determine focal plane. Automated settings were used to determine the focal plane. Spheroids were imaged on 5x focal planes covering a total of 269.5 μm on the Z-axis. Fluorescence exposure, LED intensity, integration time and camera gain were determined based on the internal control.

### Image processing and subpopulation analysis using Gen5 software

Acquired images were processed using the Gen5 software (V3.04, Biotek). Briefly, an automated pre-processing step was applied to all images to refine signal and contrast. RFP signal threshold, minimal and maximal cell size values were set to ensure individual cells were counted. To define the LC subpopulation within the total cell count, we applied a minimum GFP fluorescence signal threshold of > 2500–3000 fluorescence units (FU) across experiments. FU values were defined based on wild-type and/or unlabelled internal negative controls. The focus stacking algorithm was used for 3D outgrowth measurements, single images were generated from captured Z-stacks. Final images were then objectively measured for cellular outgrowth from the spheroid core using the cellular analysis and object mask function in Gen5 (V3.04, Biotek).

### AlamarBlue™ cell viability assays

To assess cellular viability, cells were incubated in AlamarBlue™ cell viability reagent (Invitrogen, #DAL1025) at 10% of the well volume for 6 h at 37 °C with 5% CO_2_. Fluorescence intensity was read using the Cytation™ 3 Multimode Imager at 570nm_ex_/600nm_em_ or absorbance at 570 nm and 600 nm. Cell viability was calculated based upon the measurements of replicate wells.

### Cellular apoptosis via cleaved Caspase-3 co-staining

1 × 10^5^ cells/per 24 well plate were seeded on glass coverslips incubated overnight to form a confluent monolayer. Cisplatin was added at IC^60^ concentrations determined by previous experiments for 48 h. Cells were fixed, permeabilised, blocked and stained with the Cleaved Caspase-3 antibody ASP^175^ (Abcam #ab2303) (1:2000) followed by the goat anti-rabbit IgG H&L Alexa Fluor® 647 secondary antibody (Abcam, Cambridge, UK). Coverslips were removed from the 24-well plate and were mounted onto microscope slides using FluorSave mounting medium (Calbiochem #345789). Slides were imaged using the Cytation™ 3 Multimode Imager. Subsequent subpopulation analyses were performed as described.

### xCELLigence real time cell analysis

Real time cell analyses (RTCA) were conducted using the xCELLigence RTCA SP 96-well instrument (ACEA Biosciences). Cell lines were synchronized to G_0_ by overnight incubation in serum-free media prior to commencement. For proliferation assays, cells were seeded at 0.2 × 10^4^cells/0.1 ml/well into xCELLigence E-plates (Agilent, #300601010), and impedance readings taken every 15 min for the experimental duration. Cells were treated with varying concentrations of chemotherapeutics diluted in culture medium as outlined in the experimental text. All assays were performed in duplicate or triplicate, in at least three independent experiments.

### Half maximal inhibitory (IC^50^) concentrations of cisplatin

Maximal inhibitory concentrations of drugs were assessed by AlamarBlue™ cell viability assays (Invitrogen, #DAL1025). Cells were seeded at 0.2 × 10^4^ cells/0.1 ml/well in 96-well plates, incubated for 12 h, serum starved, and then treated with concentrations of cisplatin ranging from 2.5–100 μg/ml for 48 h. Cells were imaged at regular intervals for the emergence of GFP^+^ prior to the addition of AlamarBlue™. GraphPad Prism v8.0 was used to plot data and calculate the IC^50^ using non-linear regression fits. For each assay, samples were run in triplicate or quadruplicate, and the data shown are representative of at least three independent experiments.

### Human Tissue Arrays & Immunohistochemistry

Immunohistochemistry was performed on sections obtained from High Grade Serous Epithelial Ovarian Cancer (HGS EOC) patients pre- and post-neoadjuvant chemotherapy (Southern health human ethics committee approvals HREC#02031B, #06032C). For antigen retrieval, sections were incubated for 10 min in 50 mM glycine (pH 3.5) at 90 °C. Sections were stained according to [11] with positive immunostaining assessed relative to parallel sections exposed to an isotype (IgG) control. Immunostaining in tumour and stromal tissue was assessed using Aperio ImageScope (v 12.3.3) as described [15].

### Flow cytometry for leader cell and proliferation markers

Cells were seeded at 0.3 × 10^6^ cells/well in 6-well plates, incubated for 16 h, and analysed using the BD LSRFortessa X-20 (BD Biosciences, CA, USA). Anti-human Fc block (1:50, BD Biosciences, CA, USA) was used to block non-specific Fc receptor binding. Zombie Aqua™ fixable viability dye (BioLegend, CA, USA) was used to distinguish live and dead cells. Cells were stained with Ki67-BV786 (BD Biosciences, 1:100, clone #B56) for analysis of cellular proliferation and immediately analysed. Single stained cells were used to set compensation controls, and fluorescence-minus one (FMO) controls were used to define population gates. Data was acquired using a BD LSRFortessa X-20 (BD Biosciences, CA, USA), and was analysed using FlowJo software v10.5.0 (LCC, OR, USA).

### Real time PCR

Cell were sorted by flow cytometry based upon their LC positive (GFP^+^) and follower cell (GFP^−^) status using the FACS ARIA Fusion cell sorter (BD Biosciences, CA, USA). Total RNA was extracted from the separate populations using the RNeasy Mini Kit as per the manufacturers protocol. Sense and antisense oligonucleotide primers to: *KRT14*; stemness markers: *Nanog*, *CD44v6*, *ALDHI*, *Twist*, *WNT1*; and EMT markers: *E-cadherin*, *N-cadherin*, *vimentin*, *EpCAM, fibronectin, CD133, CD117, notch, slug, snail* and *OCT4A* were designed against published human sequences and verified as previously described [16]. Primer sequences can be found in Supplementary Table 1. cDNA was synthesized using Superscript III reverse transcriptase (Life Technologies, Grand Island, NY). Real-time PCR samples were prepared to a final volume of 10 μl using the Applied Biosystems ABI SYBR mix (Scoresby, Victoria, Australia). Quantitative real time PCR was completed as previously described [17] using the Applied Biosystems ABI 7900 HT Fast real-time machine with all reactions performed in triplicate. Yields were converted to femtograms based on the standard curve for each PCR product, and the resultant mRNA levels were normalized to the 18S mRNA level per sample.

### Statistical analysis

Statistical analyses were performed on quantified data using GraphPad Prism 9 software (version 9.0), with data normalised where required. For comparison of mean values, one-way ANOVA was used to determine significance of variance (viability assay). Pairwise comparisons were performed using unpaired *t*-tests and Welch’s correction was applied (cell counts). For comparison of IC^50^ Cisplatin concentrations, viability data were log transformed, normalised and then plotted as a non-linear regression of Log[inhibitor] vs. response. Results of *p* < 0.05 were considered significant. * *p* < 0.05, ** *p* < 0.01, *** *p* < 0.001, **** *p* < 0.0001.

## Results

### Construction of a tool to monitor leader cell abundance and kinetics in vitro

**Fig. 1:**
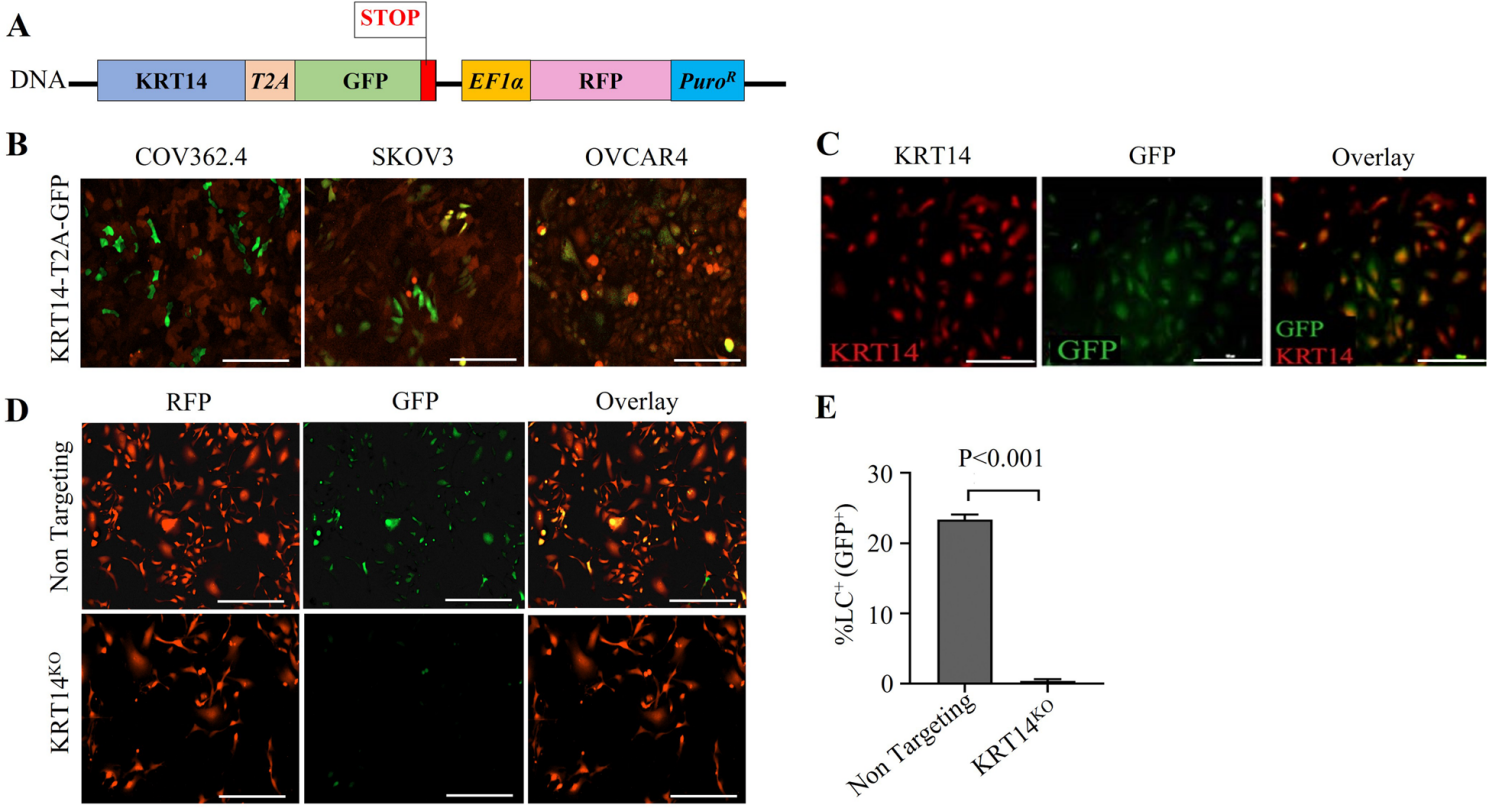
Generation of KRT14-T2A-GFP leader cell lines. **A** Schematic illustration of the KRT14-T2A-GFP construct. **B** COV362.4, SKOV3, and OVCAR4-KRT14-T2A-GFP cells, LCs marked by GFP, all cells marked by RFP. **C** KRT14-T2A-GFP lines were fixed and stained with a polyclonal KRT14 antibody and showed positive co-staining. Scale bar represents 300 μm. **D** Homozygous KRT14-T2A-GFP cells were transfected with constructs: “KRT14^KO^” - targeting exon 1 of the KRT14 gene or the “non-targeting” lentiCRISPR V1 control alone. Cells were imaged at 4X magnification for GFP and RFP 72-h post transfection. **E** LC^GFP+^ counts in KRT14^KO^ CRISPR targeted lines demonstrated a significant reduction of GFP expression compared to the non-targeting lentiCRISPR V1 control by unpaired t-test and Welch’s correction (*p* < 0.001) (*n* = 4 replicates from one representative experiment). Scale represents 100 μm

Leader cells are a functionally distinct subpopulation of ovarian cancer cells that direct cellular migration and invasion [11]. To facilitate studies of the LC subset in ovarian cancer, we used CRISPR to insert a bicistronic T2A-GFP cassette under the control of the native KRT14 promoter (Fig. 1A). In this system, the native KRT14 stop codon is removed to allow co-transcription and independent translation of KRT14 and GFP via the T2A linker sequence providing a robust and easily identifiable marker for the LC subset in vitro. Furthermore, transfected cells that successfully integrate the T2A expression cassette are marked by constitutive expression of RFP driven from an independent EF1α promoter. T2A-GFP-expressing integrants were generated using human ovarian cancer cell lines OVCAR4, COV362.4 and SKOV3. Homozygous integration of the construct was verified by genomic screens and Sanger sequencing (data not shown). Following transfection and selection, GFP could be visualized in a subset of cells consistent with the restricted expression of leader cells (Fig. 1B). Flow cytometry assessment of KRT14^+^ LC basal levels showed that they typically comprise 10–30% of the live cell population in the established cell lines examined: SKOV3, LC 27%, FC 73%; COV362.4, LC 33%, FC 77%; OVCAR4, LC 26%, FC 74% (n = 3 independent experiments).

GFP integration and expression had no apparent effect on cell viability and cell turnover with both lines maintaining similar proliferation rates (Supplementary data). The specificity of GFP expression as a surrogate marker for LCs was independently verified in two ways. First, cells were stained using an anti-KRT14 antibody, and antibody co-staining with GFP was assessed by fluorescence microscopy and flow cytometry. GFP^+^ cells were exclusively co-stained with the LC marker KRT14 (Fig. 1C), demonstrating restriction of GFP expression to the KRT14^+^ LC population. Second, CRISPR-mediated knockout of the KRT14 gene [11] (upstream of the T2A site) resulted in loss of GFP fluorescence after 72 h, with no reduction observed in cells transfected with the non-targeting control (*p* < 0.001) (Fig. 1D, E). Collectively the data demonstrate that GFP is obligately expressed with KRT14 in vitro, and provided a robust surrogate marker for the LC population.

### Leader cells do not represent a fixed lineage or quiescent cells

**Fig. 2:**
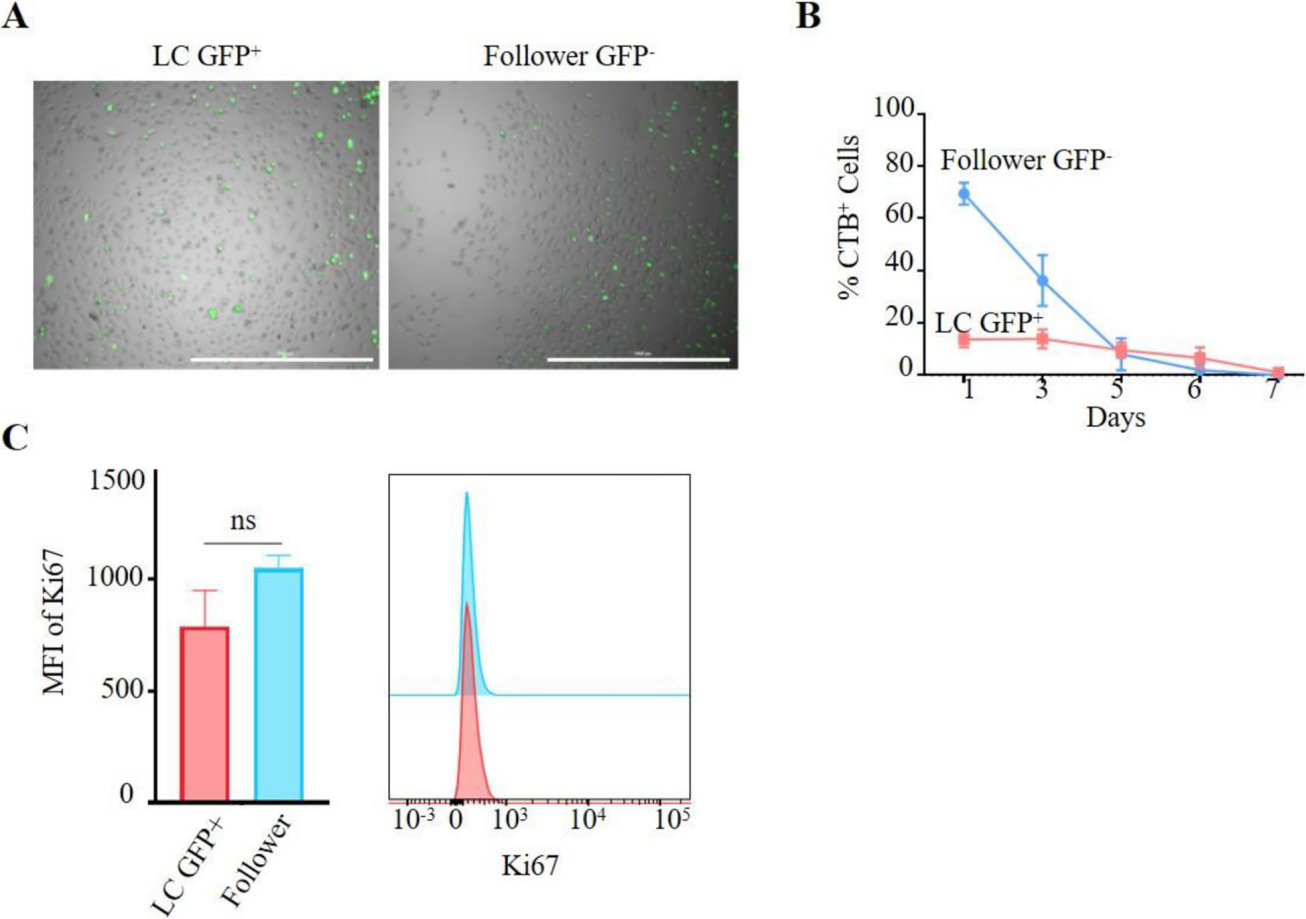
Leader cells do not represent a fixed lineage. **A** LC-T2A-GFP lines were single cell sorted based on LC^GFP+/−^ status into a single well of a half area 96-well plate and imaged daily at 4X magnification under bright field, for GFP status using the Cytation™ 3 Multimode Imager, scale represents 1000 μm. **B** LC-T2A-GFP lines were labelled with the pro-dye CellTrace™ Blue (CTB) and routinely imaged for LC^GFP+^ and CTB status over a seven-day period using the Cytation™ 3 Multimode Imager. Data are representative of duplicate experiments with 4 imaging areas per triplicate well and are presented as the mean. Representative data from COV362.4-T2A-GFP cells are shown where *n* = 3 from two experimental replicates. **C** Flow cytometric analysis of Ki67 surface expression in LC^+/−^ populations. Cells were seeded at 300,000 cells/well in a 6-well plate, incubated for 18 h, collected and stained for Ki67 using a Ki67-BV786 antibody. GFP (indicative of KRT14 expression) and BV786 fluorescence was acquired using the BD LSRFortessa™ X-20 and data was analysed using FlowJo software (v10.5.0). Analysis was performed using an unpaired non-parametric Mann-Whitney U test to determine statistical significance between groups; *n* = 2–4/cell line; one representative cell line (SKOV3) is shown (error bars indicate n = 2 replicates); ns = not significant

To assess whether the LCs represent a fixed lineage or a quiescent, stem-cell like population, GFP^+^ cells were sorted by single cell flow cytometry and monitored for LC^+^/GFP^+^ status by live cell fluorescence microscopy. For each single cell clone expanded, we observed the emergence of both GFP positive and negative cells [15], suggesting that LC status (as determined by KRT14 expression) is transient and that LCs exist as a differentiation state rather than a fixed lineage (Fig. 2A).

To identify whether LCs represent a “stem-like” or “quiescent” population, LC-T2A-GFP lines were labelled with the pro-dye CellTrace™ Blue. Quiescent cells retain the dye long-term, whilst actively dividing cells dilute the dye with each cell division [16]. The GFP^+^ / CellTrace™ Blue^+^ status of cells was monitored over a period of 7 days. We observed no significant difference in dye retention between GFP^+^ vs. GFP^−^ cell populations, indicating that the LC population does not represent a quiescent cell population (Fig. 2B).

We further examined the proliferative status of LCs vs. the bulk follower population. Cells were stained with the proliferation marker Ki67 and analysed by flow cytometry according to LC GFP^+^/GFP^−^ status. There was no significant difference in Ki67^+^ status between LC^+^GFP^+^ vs. follower GFP^−^ cells, suggesting no observable difference in the rate of cellular turnover and proliferative state between LCs and the follower population (Fig. 2C).

### Chemotherapy does not inhibit LC function and leads to their enrichment in vitro

Increased expression of KRT14 is negatively associated with progression-free survival and response to therapy in ovarian cancer patients [11], and KRT14^+^ cells are also enriched in chemo-resistant bladder cancer [12]. Utilizing our T2A-GFP cell lines we examined the response of LCs against a panel of chemotherapeutics commonly used to treat ovarian cancer, including olaparib, topotecan, rucaparib, cylcophosphamide, doxorubicin, carboplatin, cisplatin and paclitaxel. Doses were defined based upon established literature and ranged from 100 nM-1 μM, with inhibitory concentrations empirically confirmed by independent dose response experiments as indicated in the figure legends. Following incubation with each drug, the total (RFP^+^) and LC (GFP^+^) cell populations were assessed. All chemotherapeutics tested reduced overall cell viability as expected, with paclitaxel and doxorubicin achieving the greatest reduction in cell viability (Table 1). However, concurrent fluorescence imaging revealed a proportionate increase in the GFP^+^ LC population under each treatment condition, consistent with the enrichment of LCs following exposure to chemotherapy (Table 1).

**Table 1:**
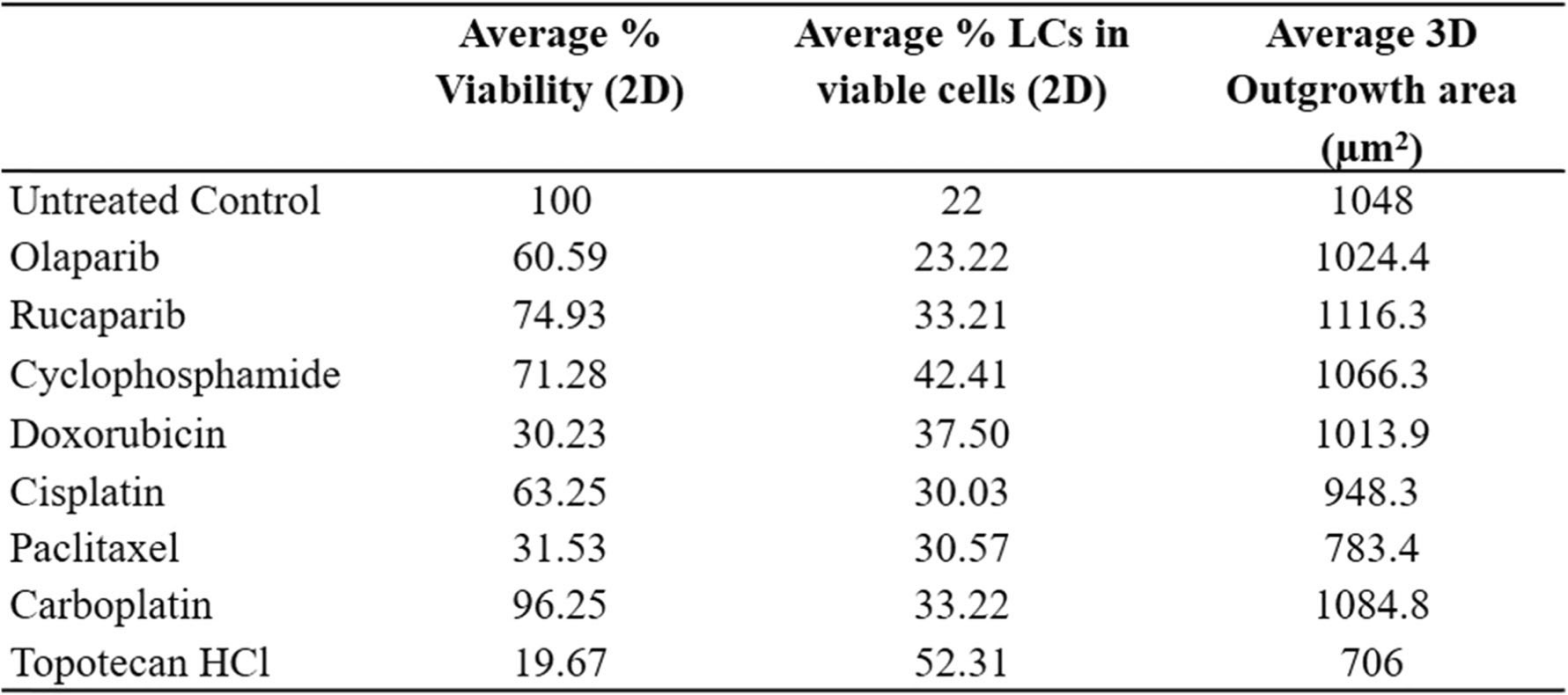
The effect of common chemotherapeutics on cell viability, Leader cell population and the migration of ovarian cancer cells

LC-T2A-GFP cells were seeded as monolayer or spheroids and subsequently treated with chemotherapeutics including olaparib, rucaparib, cisplatin, cyclophosphamide, doxorubicin, paclitaxel, carboplatin and topotecan for 72 h. Following treatment, cells were imaged on the RFP/GFP channels to assess LC status. Spheroid outgrowth was measured using the Cytation™ 3 Multimode Imager and analysed using the Gen5 software (V3.04, Biotek). Cells were incubated with 10% alamarBlue™ reagent for 6 h and fluorescence intensity/absorbance read. Percentage viabilities were calculated based on untreated control wells and the no-cell internal control. The data provided are representative average values from four independent experiments and 2 replicates per experiment.

**Fig. 3:**
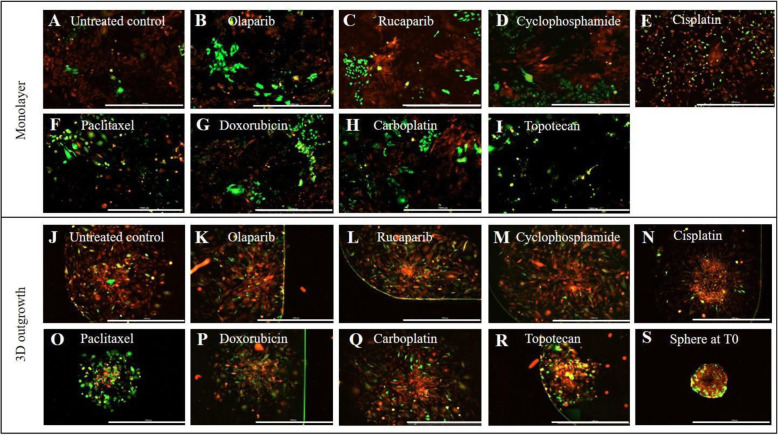
Ovarian cancer Leader cells are enriched in response chemotherapeutics commonly used to treat disease. LC-T2A-GFP cells were seeded as a: **A-I** monolayer; 10,000 cells/single well of a 96-well plate adhered and serum starved overnight or **J-S** spheroids; 2500 cells/single well in 96-well ultra-low adhesion plates; once formed, spheres were transferred into 96-well flat-bottom imaging plates using wide bore tips. Cells were subsequently treated with olaparib (**B, K**), rucaparib (**C, L**), cyclophosphamide (**D, M**), cisplatin (**E**, **N**), paclitaxel (**F, O**), doxorubicin (**G, P**), carboplatin (**H, Q**), and topotecan (**I**, **R**) in spheroid and monolayer format for 72 h, with untreated controls (**A, J**) and a representative spheroid at assay commencement shown (**S**). 72 h post treatment, duplicate wells were imaged on RFP and GFP channels at 4X magnification to determine LC^GFP+^ status and spheroid outgrowth using the Cytation™ 3 Multimode Imager. Spheroids were imaged on 5 focal planes (Z-stacked) and analysis was performed on focus-stacked images. Scale bar 1000 μm

Importantly, the LCs represented the bulk of the remaining cell population following treatment (Fig. 3A-I), demonstrating their inherent resistance to chemotherapy. Our previous work also demonstrated that LCs are essential for spheroid attachment, displacement and invasion of the mesothelial cell layer [11]. Consistent with their survival in the presence of chemotherapy drugs, LC migration in 3D spheroid outgrowth assays were unaffected in the presence of all but one of the chemotherapies tested (Fig. 3J-R). Topotecan represented the only drug that exhibited inhibition of outgrowth of the highly invasive and migratory LC population (Fig. 3I, R), however, topotecan enriched the LC subset 20% above the untreated control level (Table 1).

**Fig. 4:**
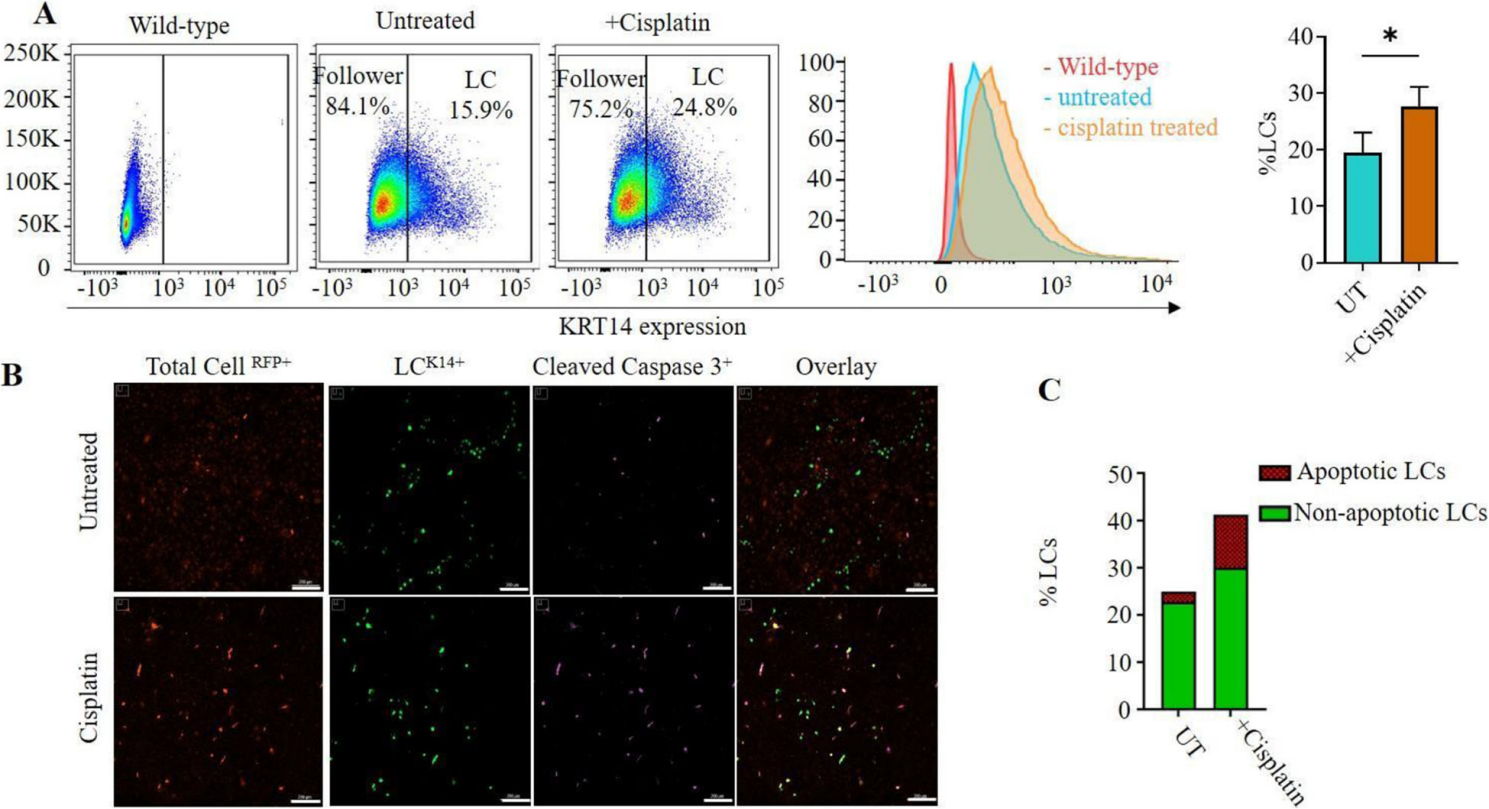
Ovarian cancer Leader cells represent a population of cells resistant to chemotherapy induced apoptosis. **A** LC-T2A-GFP cells were treated with the IC^50^ dose of cisplatin and sorted by flow cytometry. Cells were gated based on positive and negative controls where cisplatin treatment increased the percentage of the GFP^+^ LCs significantly. LC and FC percentages are expressed as the percentage of live cells. **B** LC-T2A-GFP cells were seeded as a monolayer on glass coverslips and treated with IC^50^ concentrations of cisplatin for 48 h. Cells were fixed and stained with the apoptosis maker, Cleaved Caspase-3 and imaged for GFP, Cleaved Caspase-3 and DAPI using the Cytation™ 3 Multimode Imager. **C** Data was analysed using the Gen5 software (V3.04, Biotek) to calculate the total cell counts, percentage of LCs (GFP^+^), apoptotic cells (cleaved caspase-3^+^) and double positive cell numbers. Statistical analysis was performed using a paired t-test; n = 4 focal areas per well. Scale bar at 200 μm

To further characterise the mechanism of underlying drug resistance in LCs, we evaluated cisplatin-induced apoptotic cell death by staining for cleaved caspase-3. Flow cytometry confirmed an increase in the percentage of LCs following treatment with cisplatin (Fig. 4A) (Live cell imaging of GFP enrichment - supplementary material). Through fluorescent imaging we observed low to no apoptotic cells in untreated cell lines, with the percentage of GFP^+^ LCs reflective of the basal amounts observed in previous experiments. Following treatment with an IC^50^ dose of cisplatin for 72 h, we observed the percentage of GFP^+^ LCs to be significantly increased compared to the untreated control for all conditions examined (Fig. 4B). As expected, chemotherapeutic intervention induced a significant increase in the number of cleaved caspase − 3 positive cells potentially indicating increased apoptosis. Further Cleaved caspase-3 cells were almost exclusively LC negative (Fig. 4C).

### KRT14 expression is enriched in neoadjuvant chemotherapy treated specimens

**Fig. 5:**
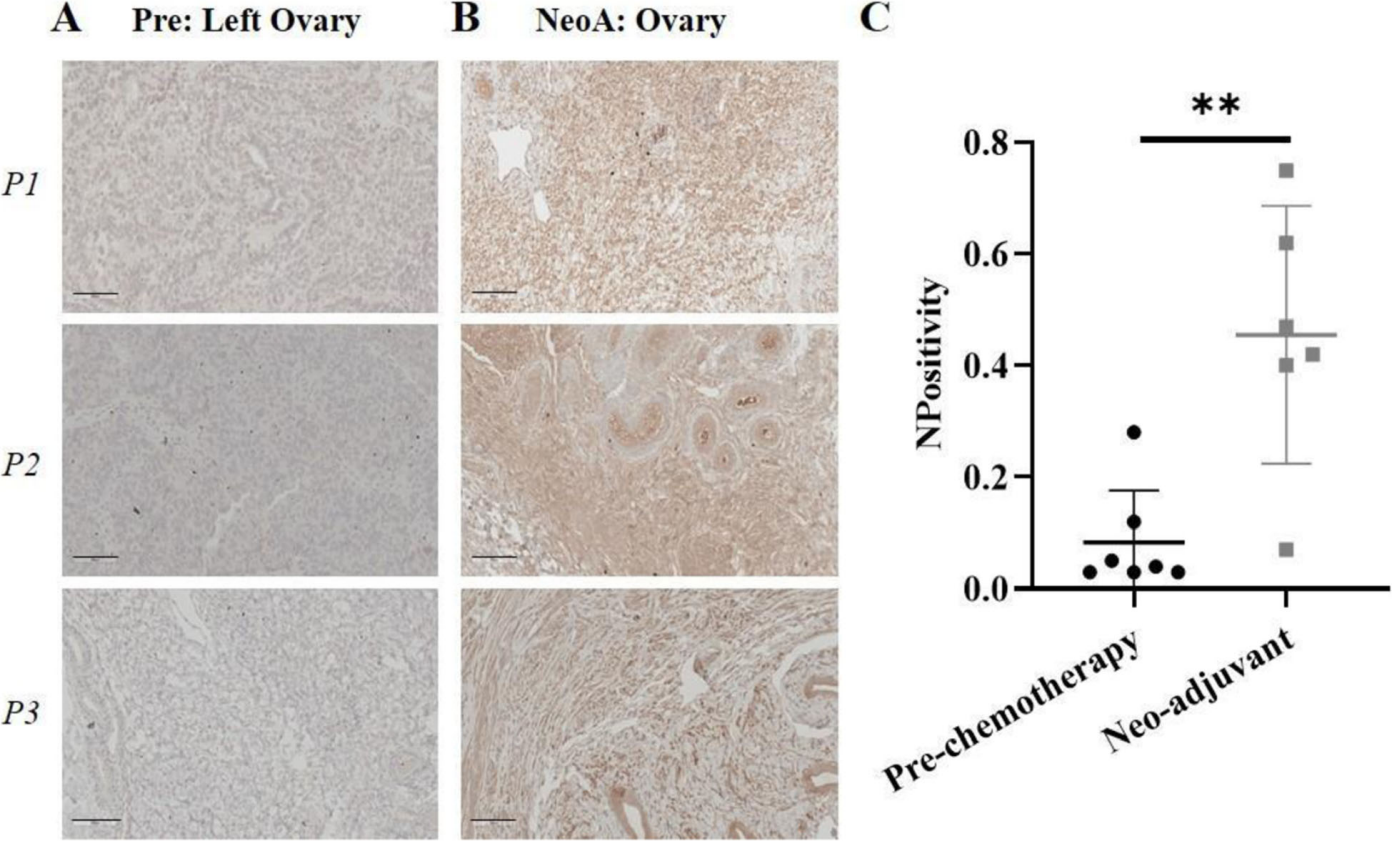
Representative immunohistochemical staining of KRT14 in **A** HGS ovary pre-treatment and **B** neo adjuvant ovary at 20X magnification, scale 100 μm. **C** Positive pixel staining count (*p* = 0.0047, Mann-Whitney U test)

To establish the clinical relevance of KRT14 expression in response to chemotherapeutics, we determined abundance and localization of KRT14 in OC cells from HGS EOC patients receiving neoadjuvant chemotherapy by immunostaining. In agreement with our previous findings, KRT14 staining was localized to tumor epithelium, with little evidence of KRT14 in stromal tissue (Fig. 5A). KRT14 staining was significantly elevated in all neoadjuvant treated specimens compared to chemo-naïve samples (Fig. 5B) (*p* = 0.0047, Mann-Whitney U test).

### Chemotherapy-induced leader cells arise independent of cellular division or DNA replication

**Fig. 6:**
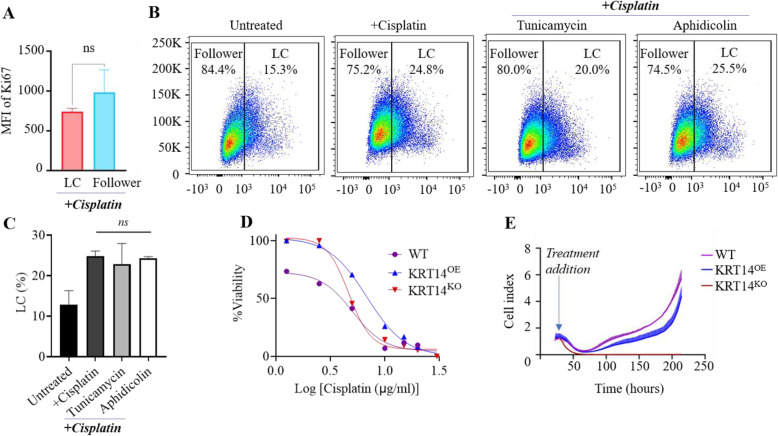
Leader cells proliferate at the same rate in chemotherapy and do not arise due to cellular division and DNA replication, leader cell deficient lines are more susceptible to cisplatin treatment. **A** Flow cytometric analysis of Ki67 surface expression in LC^+/−^ populations in ovarian cancer cell lines in the presence of cisplatin. **B** Cells were seeded at 300,000 cells/well in a 6-well plate, incubated for 18 h, and treated with 13 μg/ml cisplatin +/− aphidicolin (0.5 μg/ml) or tunicamycin (6 μg/ml) for 24 h. Following treatment, cells were collected and stained for Ki67 using a Ki67-BV786 antibody. GFP (indicative of KRT14 expression) and BV786 fluorescence was acquired using the BD LSRFortessa™ X-20 with data analysed using the FlowJo software (v10.5.0). **C** Analysis was performed using a non-parametric Mann-Whitney U test to determine statistical significance between groups; *n* = 2–4/cell line; ns = not significant. **D** Wild-type, LC-deficient (KRT14^KO^) and LC-enriched (KRT14^OE^) cells were seeded at 0.2 × 10^4^ cells/0.1 ml/well in 96-well plates. Cells were synchronized in G_0_ by overnight incubation in serum-free media prior to treatment and treated with cisplatin concentrations ranging from 1.2–30 μg/ml for 48 h followed by the addition of cell viability dye alamarBlue™ (Invitrogen). Half maximal inhibitory (IC^50^) concentrations of cisplatin were calculated in GraphPad Prism (v9.0) and analysed by non-linear regression fit. **E** Long-term assessment of cellular proliferation in response to high doses of Cisplatin where 0.2 × 10^4^ cells/0.1 ml/well wild-type, LC deficient (KRT14^KO^) and LC enriched (KRT14^OE^) cells were seeded onto RTCA xCELLigence E-plates and treated with 30 μg/ml cisplatin, cellular proliferation was monitored over 240-h and impedance readings taken every 15 min

Whilst we observed no difference in proliferation between LCs vs. follower cells, it is well-established that cells with slower proliferative rates are typically less susceptible to chemotherapy [18]. We therefore evaluated proliferation of LCs vs. followers in the presence of cisplatin using Ki67 staining and flow cytometry. There was no significant difference in Ki67^+^ status between LCs and followers (Fig. 6A). To assess whether DNA replication and/or cell division were required for LC status, the relative proportion of cisplatin-induced GFP^+^ LCs was evaluated by flow cytometry in the presence of aphidicolin or tunicamycin (to inhibit DNA replication or cell division respectively). As previously observed, cisplatin treatment increased the proportion of LCs; neither tunicamycin nor aphidicolin had any discernible effect on the percentage of LCs compared to the cisplatin-treated sample alone (Fig. 6B, C).

The demonstrated chemo-resistant nature of LCs strongly suggests a role in acquired chemo-resistance in vivo. Chemo-responsiveness to cisplatin (0-30 μg/ml) was evaluated in wild-type, KRT14^KO^ (i.e. LC-deficient) and KRT14^OE^ (i.e. LC-enriched) cell lines [11] using xCELLigence RTCA. LC-deficient (KRT14^KO^) cells had greater sensitivity to chemotherapy (IC^50^: 4.674 μg/ml) vs. the wild type control (IC^50^: 5.046 μg/ml); by contrast, LC-enriched cells (KRT14^OE^) had reduced sensitivity (IC^50^: 6.830 μg/ml) (Fig. 6D). Long-term cell viability and recovery following high-dose cisplatin (30 μg/ml) exposure was also assessed. Both the wild-type and LC-enriched (KRT14^OE^) cells recovered following an initial decline in proliferation, suggesting repopulation and recovery by the more chemo-resistant LCs (Fig. 6E). By contrast, LC-deficient KRT14^KO^ cells failed to recover following treatment demonstrating their greater susceptibility to chemotherapeutic intervention thus further suggesting LCs are required for tumour re-population.

### Leader cells co-express known chemo-resistance markers and have a stemness profile that is not correlated with EMT

**Fig. 7:**
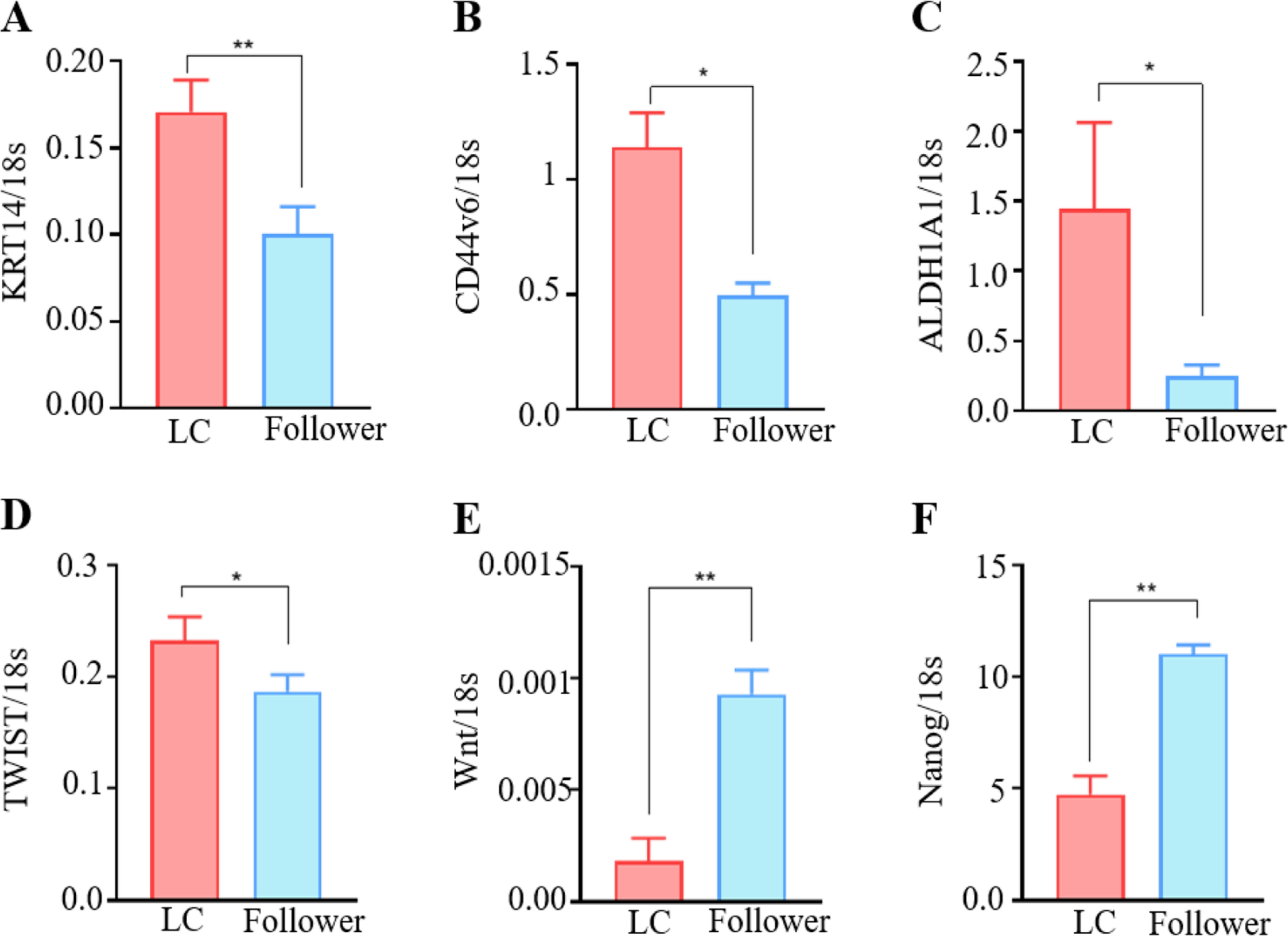
Leader cells express markers indicative of a “stemness” profile. Cells were sorted by flow cytometry based upon LC status, mRNA was extracted and cDNA synthesized. Gene expression of **A** KRT14, **B** CD44v6, **C** ALDHI, **D** Twist, **E** WNT and **F** Nanog was analyzed by quantitative real-time PCR. Data was normalised to ribosomal 18S and graphed as the mean value ± SEM with a single representative real time experiment shown. Analysis was performed by one-way ANOVA and Tukey’s multiple comparison post hoc test to determine statistically significant differences between groups (* *p* < 0.05, ** *p* < 0.01) (*n* = 3 separate isolations in a single run)

Previous studies in prostate, breast and bladder cancer demonstrate that LCs may be associated with chemo-resistance and express markers associated with a “cancer stem cell” population [12, 19]. Flow cytometry was used to isolate GFP^+^ LCs from the follower population, with *KRT14* expression confirmed by qRT-PCR (Fig. 7A). We examined the relative expression levels of several genes previously associated with “stemness” and / or epithelial-to-mesenchymal transition by qRT-PCR in the two cell populations. Key “stemness” markers including *CD44v6*, *ALDHI* and *Twist* were all increased in the LC population; whilst *WNT* and *Nanog* were decreased (Fig. 7B-F). Interestingly, there were no significant differences between the two populations for the EMT markers *E-cadherin*, *N-cadherin*, and *Vimentin*; nor for *EpCAM*, *fibronectin*, *CD133*, *CD117*, *Notch*, *Slug*, *Snail* or *OCT4A (Supplementary data)*.

## Discussion

Disease relapse and acquired resistance pose the most significant challenge to the effective management of ovarian cancer. We recently have identified an ovarian cancer LC subset responsible for mediating the invasive and migratory propensity of ovarian cancer cells, which is also strongly associated with reduced progression-free survival post-chemotherapy [11, 12, 20]. Our new data expands upon this role, indicating that the ovarian cancer LC subset may contribute significantly to chemo-resistance and disease progression in vivo.

Exposure to several commonly prescribed chemotherapy agents, each of which exert their anti-neoplastic activity via independent mechanisms, resulted in significant enrichment of the ovarian cancer LC population in vitro. Whilst untreated cell lines exhibited a LC proportion of ~ 10–30% in culture, 7 of 8 chemotherapies examined directly increased LC proportions to above 20% after 72 h. Longer-term culture also demonstrated the recovery and renewed proliferation of both wild-type and LC^+^/KRT14^+^ cells after cisplatin exposure, whereas cells lacking KRT14 expression failed to recover. This result is also in agreement with a previous study in bladder cancer, where repeated tamoxifen chemotherapy increased the LC^+^/KRT14^+^ cell population in vivo [12]. Cell populations enriched for KRT14 also exhibited increased IC^50^ values following cisplatin therapy compared to wild-type or KRT14^KO^ cells and were less susceptible to apoptotic death, suggesting chemo-resistance may be mediated in part via a KRT14-dependent mechanism, and suggests that KRT14 overexpression may contribute in part to acquired chemo-resistance in ovarian cancer patients with the data suggesting that LC abundance is closely related to chemo sensitivity. However, neither overexpression nor ablation of *KRT14* altered proliferation, viability or cellular turnover in vitro, indicating KRT14 likely plays an indirect role in this process.

Self-renewal and the resumption of cancer cell proliferation following treatment is an important characteristic of cancer recurrence [21], and the quiescent, slow-cycling cancer stem cell population is believed to drive clinically acquired chemo-resistance in patients [22]. The self-renewal and multipotent capabilities of LCs have previously been suggested in multiple cell types [12, 23–25]. However, our study demonstrates that ovarian cancer LCs do not represent a quiescent population in vitro, with no difference in proliferation rate observed between LCs and follower cells. Moreover, the kinetics of LC cycling did not correlate with their susceptibility to chemotherapy. Nevertheless, LCs gave rise to both KRT14^+^ and KRT14^−^ daughter cells, indicating that LCs may exist as a non-terminally differentiated phenotype in ovarian cancer. The bi-directional state of differentiation also suggests that similar to breast cancer [9, 10], LCs represent a transcriptionally plastic differentiation state rather than a fixed lineage.

Ovarian cancer LCs expressed some, but not all, genes commonly associated with EMT and stem-like behaviour. Whilst EMT markers *E-cad*, *N-cad* and *Vimentin* remained unchanged, expression of *CD44v6*, *ALDH1* and *Twist* were all elevated in LCs with respect to the follower population. Both ALDH1 and Twist have previously been associated with stemness and EMT-like features in circulating tumour cells (CTCs) from breast cancer patients [26]. The ALDH1^+^ Twist^+^ CTCs were chemo-resistant, correlated with lung metastases and were an independent predictor of poor prognosis [26]. Both KRT14 and Twist expression have also been identified as drivers of collective invasion and markers of breast cancer micro-metastases [9, 10, 27, 28]. Furthermore, CD44v6 is associated with invasion, metastasis and chemo-resistance in vitro in prostate cancer cells [29]. Our data therefore suggests that ovarian cancer LCs retain features of both stemness and EMT that contribute to their enhanced malignant potential.

## Conclusions

Our observation that standard chemotherapy agents enriched the aggressive, invasive and chemo-resistant LC subpopulation in vitro, and the enhanced percentage of LCs when comparing KRT14 staining in tissues collected from HGSOC patients prior and after chemotherapy, provides a plausible explanation for the high rate of relapse and emergent drug resistance amongst ovarian cancer patients. These findings have important implications in a clinical setting; we show LCs are necessary for tumour re-population following chemotherapy with LC enrichment contributing to increased aggression/resistance collectively suggesting LCs serve as an attractive therapeutic target. Therapies specifically targeting the LC subset in tumours are likely to provide robust, long-lasting tumour regression for patients with recurrent or drug-resistant disease.

## Supplementary Information

**Additional file 1.**

## Data Availability

All data generated or analysed during this study are included in this published article [and its supplementary information files].

## Abbreviations

LC: Leader Cell
CSC: Cancer Stem Cell
EMT: Epithelial to Mesenchymal Transition
KRT14: Keratin 14
HGSOC: High Grade Serous Ovarian Cancer
EOC: Epithelial Ovarian Cancer
HA: Homology Arm
GS: Guide Strand
GFP: Green Fluorescent Protein
RTCA: Real Time Cell Analyses
CTCs: Circulating Tumour Cells
FU: Fluorescent Units
CTB: Cell Trace Blue
FCS: Fetal Calf Serum
DMEM: Dulbecco’s Modified Eagle Medium
