# Supplementary material for: Chemoresistance is mediated by ovarian cancer leader cells in vitro
Source: J Exp Clin Cancer Res. 2021 Sep 1;40:276. doi: 10.1186/s13046-021-02086-3 (PMC8408956; doi:10.1186/s13046-021-02086-3)
Supplement: Supplementary file 1 — Additional file 1. [file 13046_2021_2086_MOESM1_ESM.docx]

**Table S1. RT-PCR Primer sequences.**

| **Gene** | **Primer sequence (5′-3′)** |
| --- | --- |
| *KRT14* F | TTCTGAACGAGATGCGTGAC |
| *KRT14* R | GCAGCTCAATCTCCAGGTTC |
| *Nanog* F | CAACCAGACCCAGAACATCC |
| *Nanog* R | TTCCAAGGCAGCCTCCAAG |
| *CD44v6** F | AAGGTGGAGCAAACACAACC |
| *CD44v6** R | TCCACTTGGCTTTCTGTCCT |
| *ALDH1* F | TGTTGTATAGCCGCATCCAG |
| *ALDH1* R | CCCCTTCTTTCTTCCCACTC |
| *Twist* F | TCCGCGTCCCACTAGCA |
| *Twist* R | AGTTATCCAGCTCCAGAGTCTCT |
| *WNT-1* F | CAGGAGGTTACAGGGCAAAA |
| *WNT-1* R | ACATCCAAACTCGTGGCTCT |
| *E-cadherin* F | GGCACAGATGGTGTGATTACAG |
| *E-cadherin* R | GTCCCAGGCGTAGACCAAGAAA |
| *N-cadherin* F | AAACAGCAACGACGGGTTAG |
| *N-cadherin* R | CTTAGGATTGGGGGCAAAAT |
| *Vimentin* F | CCTACAGGAAGCTGCTGGAA |
| *Vimentin* R | GGTCATCGTGATGCTGAGAA |
| *EpCAM* F | CGTCAATGCCAGTGTACTTCAGTTG |
| *EpCAM* R | TCCAGTAGGTTCTCACTCGCTCAG |
| *FN-1* F^**^ | GAGTCAGCCTCTGGTTCAGACT |
| *FN-1* R^**^ | GCAGTGTCTTCTTCACCATCAG |
| *CD133* F | CCTGGGGCTGCTGTTTATT |
| *CD133* R | ACCTGGTGATTTGCCACAA |
| *CD117* F | GATTCCCAGAGCCCACAATA |
| *CD117* R | TCTTGCCCACATCGTTGTAA |
| *Notch 1* F | AACTGCCAGACCAACATCAAC |
| *Notch 1* R | AGCTCTCATAGTCCTCGGATTG |
| *Slug* F | TCCTGGTCAAGAAGCATT |
| *Slug* R | GAGGAGGTGTCAGATGGA |
| *Snail* F | CACATCCGAAGCCACA |
| *Snail* R | AGAAGGTCCGAGCACA |
| *OCT4A^***^* F | CTCCTGGAGGGCCAGGAATC |
| *OCT4A^***^* R | CCACATCGGCCTGTGTATAT |

***** CD44V6 Transcript variants 1, 2, 3, 4, 6, and 7

****** FN1 mRNA transcript variants 1, 3, 4, 8, 13, 15, and 19

******* Transcript variant 1

**Video S1. LC enrichment in response to cisplatin treatment.**

COV362.4-LC-T2A-GFP cells were treated with 2.5 µg/mL cisplatin and imaged on RFP (total cells) and GFP (LCs) fluorescent channels for 24 hours with 30 minutes intervals to monitor cellular response in real time. A kinetic video was generated using the Gen5 software (V3.4) bundled with the Cytation3™ imaging system.


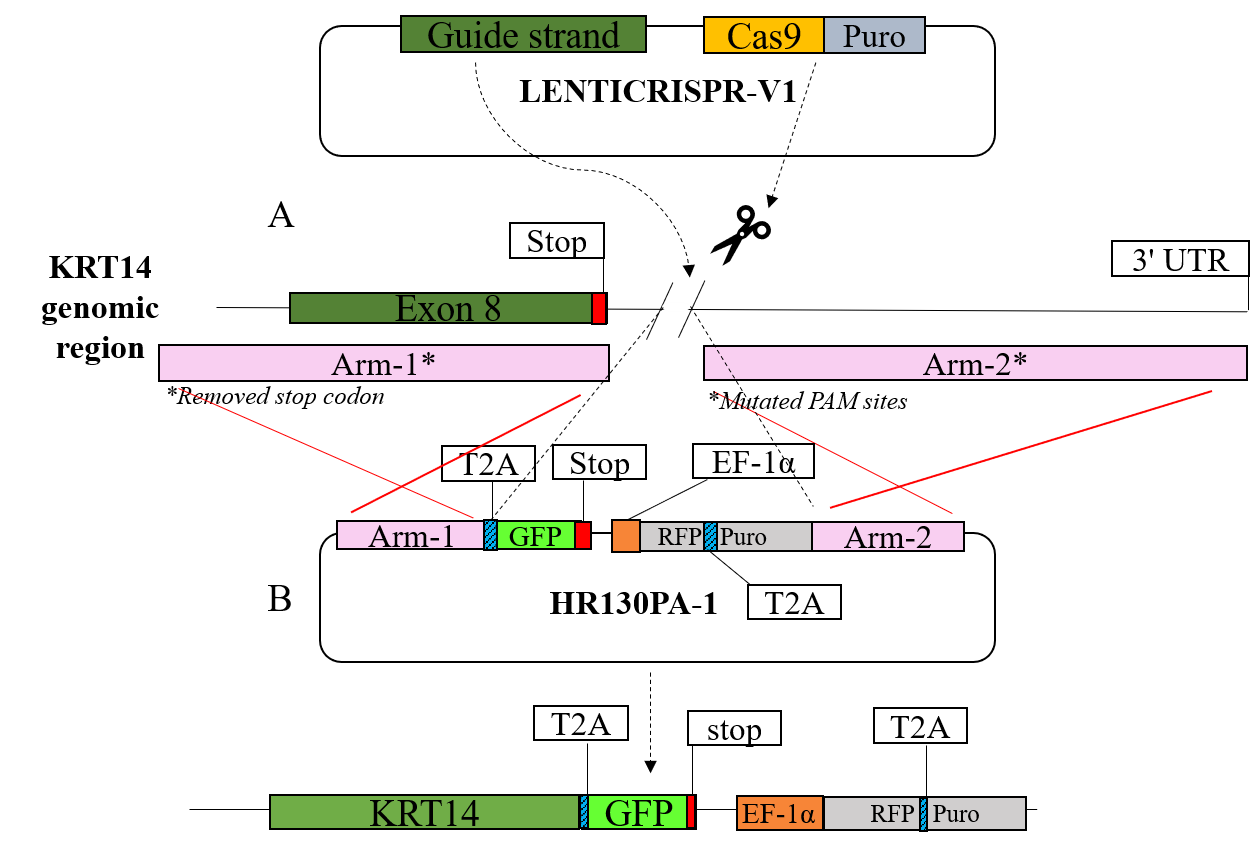


**Supplementary FIG S1. Schematic illustration of generating KRT14-T2A-GFP cell lines.** A two-vector CRISPR strategy was used. The vectors included (i) pLentiCRISPRV1 cloned with guide strands targeting 3’-UTR region of KRT14 genomic sequence and (ii) HR130PA-1 carrying homology arms complementary to KRT14 native sequence (arm 1) as well as 3’-UTR region (arm 2) with the T2A-GFP-EFIa-RFP-Puro. Cells were co-transfected with the two plasmids and were cultured in puromycin-based selection media. The T2A-GFP cells are all marked with RFP expression while only the KRT14-expressing LCs express GFP.


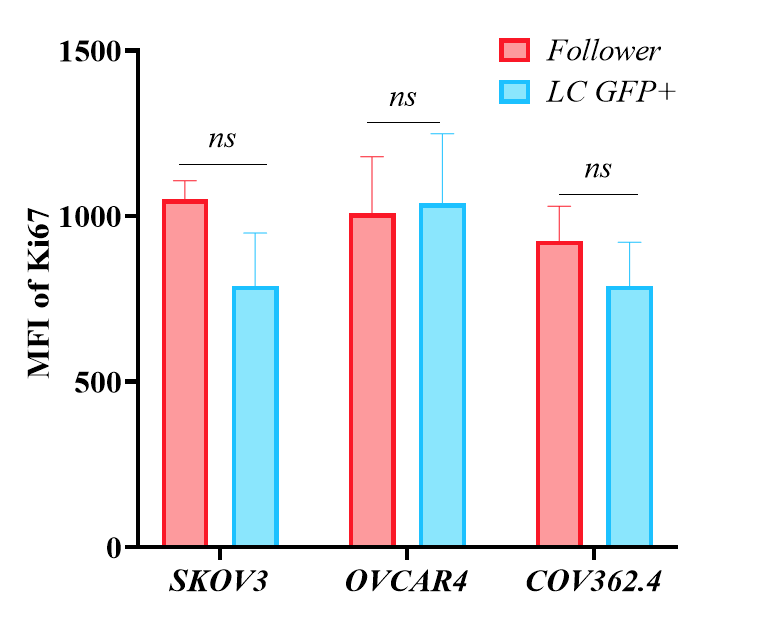


**Supplementary FIG S2.** *Flow cytometric analysis of Ki67 surface expression in LC^+/-^ populations. Cells were seeded at 300,000 cells/well in a 6-well plate, incubated for 18 hours, collected and stained for Ki67 using a Ki67-BV786 antibody. GFP (indicative of KRT14 expression) and BV786 fluorescence was acquired using the BD LSRFortessa™ X-20 and data was analysed using FlowJo software (v10.5.0). Analysis was performed using an unpaired non-parametric Mann-Whitney U test to determine statistical significance between groups; n=2-4/cell line; ns=not significant.*

*
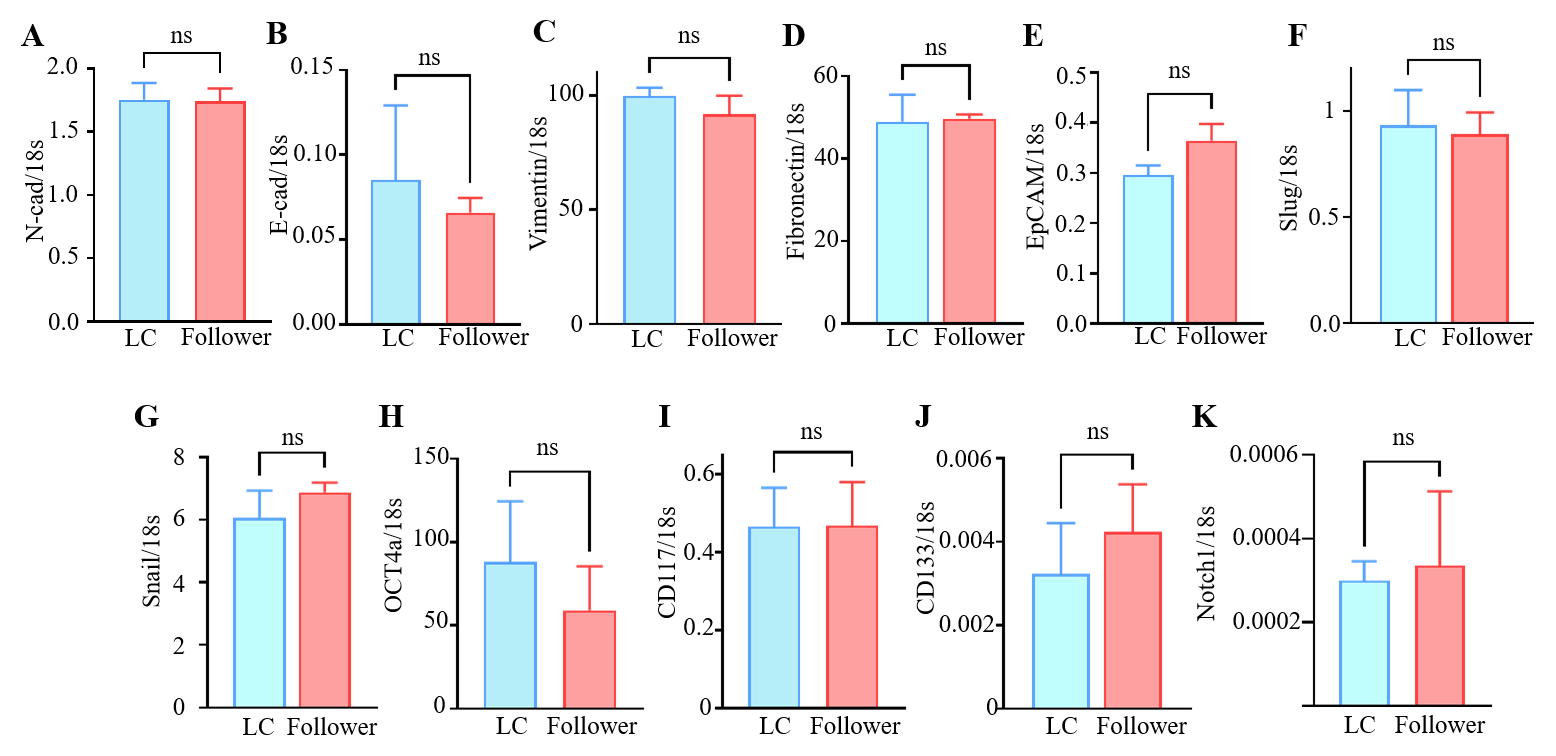
*

**Supplementary FIG S3.** Leader cells express comparable levels of markers associated with EMT and “stemness” profile. SKOV30T2A-GFP cells were sorted by flow cytometry based upon LC status, mRNA was extracted, and cDNA synthesized. Gene expression of **A)** *N-cadherin*, **B)** *E-cadherin*, **C)** *Vimentin*, **D)** *Fibronectin*, **E)** *EpCAM*, **F)** *Slug*, **G)** *Snail*, **H)** *OCT4A*, **I)** *CD117*, **J)** *CD133*, and **K)** *Notch1* was analyzed by quantitative real-time PCR. Data was normalised to ribosomal 18S and graphed as the mean value ± SEM with a single representative real time experiment shown. Analysis was performed by one-way ANOVA and Tukey’s multiple comparison test to determine statistically significant differences between groups (ns = not significant) (n=3 separate isolations in a single run).
